# Large-scale flow field and aerosol particle transport investigations in a classroom using 2D-Shake-The-Box Lagrangian Particle Tracking

**DOI:** 10.1016/j.heliyon.2023.e22826

**Published:** 2023-11-27

**Authors:** Tom Buchwald, Gazi Hasanuzzaman, Sebastian Merbold, Daniel Schanz, Christoph Egbers, Andreas Schröder

**Affiliations:** a Chair of Image Based Measurement Techniques, Brandenburg University of Technology Cottbus-Senftenberg, Cottbus, Germany; b Department of Aerodynamics and Fluid Mechanics, Brandenburg University of Technology Cottbus-Senftenberg, Cottbus, Germany; c Department of Experimental Methods, Institute of Aerodynamics and Flow Technology, German Aerospace Center (DLR), Göttingen, Germany

**Keywords:** Shake-the-box, Lagrangian-particle-tracking, Aerosol particle spreading, Room ventilation, Covid-19

## Abstract

Infections with COVID-19 in enclosed public spaces, where virus-laden aerosol particles can accumulate over time, have significantly contributed to the rapid spread of the virus. It is therefore of great importance to understand the transport and dispersion process of aerosol particles in such spaces, especially against the background of future pandemics. In this work, we present a Lagrangian-Particle-Tracking experiment to assess the mixed convective flow in a classroom with different ventilation strategies. For this purpose, thermal plumes were created by heated dummies, and a collimated LED light-sheet with ∼0.4 m thickness was used for illumination of helium filled soap bubbles (HFSB) acting as passive tracer particles. In this way, the Lagrangian trajectories of the particles were recorded at two approximately 4.2 m × 2.8 m large fields using the novel 2D-Shake-The-Box-Method. As a result, time-resolved trajectories of over 300,000 simultaneously tracked HFSB have been reconstructed, so that both small-scale and large-scale properties of the flow are visualized quantitatively across the entire cross-section of the room. The trajectories show that the thermal plumes create lengthwise circulating vortices, which cannot be destroyed across the entire cross-section of the room by opening or tilting a window. Furthermore, the mixing in the room through the operation of an air purifier is higher compared to opening a window, which suggests that this strategy in combination with its air filtering capability is the most effective strategy to prevent infections.

## 1 Introduction

The COVID-19 pandemic and its containment measures had a dramatic impact on all societies of the world. It has become clear that a detailed understanding of the virus and its transmission modes are essential for choosing appropriate protective measures. This knowledge enables avoiding ineffective or inefficient mitigation strategies. One example of this is the fact that to date, no convincing evidence has been found to suggest that smear infections via fomites play a significant role in the transmission of COVID-19 [1,2]. Accordingly, regular disinfection of surfaces does not seem to be an appropriate protective strategy. Instead, measures to contain COVID-19 should primarily be directed against airborne transmission. Since the size distribution of emitted particles as well as their viral load depend strongly on the individual and on the activity carried out, it is challenging to identify a specific particle diameter range responsible for the majority of infections. However, a growing number of studies show that aerosol particles instead of larger droplets are the main route of transmission for COVID-19, particularly in poorly ventilated closed rooms [[3], [4], [5]]. The reason for this is that droplets settle down in the order of seconds or less, while aerosol particles of a diameter between 40 μm and 100 μm typically shrink due to evaporation before they reach the floor and can linger in the air for minutes. Aerosol particles between 200 nm and 40 μm, which are emitted in the larynx, in a smaller part in the bronchioles [6] or in the alveolar ducts, even remain airborne for hours. It has been shown that the liquid phase of droplets smaller than ∼30 μm evaporates under typical indoor conditions during the time span between exhalation and settling down to the floor [[7], [8], [9]]. The remaining diameter of the aerosol particle can easily shrink to less than 5 μm in this process [10] and thus, it becomes an almost passive tracer particle at low Stokes numbers within the typical flow regimes in enclosed spaces. Therefore, virus-laden aerosol particles exhaled by infected individuals can accumulate in these environments over time. People can be infected immediately by inhaling high concentrations of the accumulated, long-lived aerosol particles or by accumulating a critical number over a longer time span lingering inside the room. That means that enclosed public spaces like churches, schools and offices play a crucial role in the spread of the virus [11,12]. Consequently, it is important to recognize that airborne transmission via small particles which plays a more important role than large droplets be considered when deciding what measures to take in closed spaces.

An essential part of the containment strategy in the past and ongoing COVID-19 pandemic is wearing masks. It has been demonstrated [13] that simple surgical or homemade masks do not provide effective protection against small droplets with a diameter of 0.3–2 μm. Nevertheless, these masks protect other people because aerosols and droplets are not transported as far away when speaking, (heavy) breathing or coughing [14]. However, the work of [15] showed that such masks still provide self-protection against an infection when aerosol concentration is limited. This is because the infection probability increases nonlinearly with the number of virus-laden particles to which a person is exposed. Since masks with limited filtering effect nevertheless result in a fractionally lower number of inhaled particles, they thus lead to a significant reduction in infection likelihood. Therefore, infections can be prevented in enclosed public spaces as long as adequate ventilation is provided and masks are worn.

Inadequate ventilation has also been shown to lead to an increase in infections with other airborne pathogens such as measles, tuberculosis [16] or rhinovirus [17]. Consequently, a quantification of minimum ventilation required for the prevention of infection is needed not only for the current COVID-19 pandemic, but also for already existing airborne pathogens and those arising in the future. However, infections can occur despite a high air exchange rate, if the ventilation carries aerosol particles from one infected person directly to the next, as shown by Refs. [18,19] in real case studies. The common well-mixed hypothesis [20] does not seem to apply in such situations. Thus, the prevention of infections depends not only on the chosen air exchange rate, but also on the flow topology and mixing inside the room and the respective transport routes of the exhaled aerosol particles. Previous experimental and CFD studies confirm that an increased risk by certain ventilation strategies can result from the creation of an unfavorable flow topology. One example is described in Ref. [21], in which different ventilation strategies were compared in a classroom situation. The simulations suggested that opening a window located directly behind a row of people carries a significantly higher hazard than predicted by the well-mixed hypothesis.

A common strategy to evaluate the risk of infection due to airborne transmission of virus-laden particles is the local concentration measurement of tracer-particles [[22], [23], [24]] or tracer gases [25]. However, these experimental approaches are limited to a few representative scenarios and do not provide any information about the Lagrangian transport routes or flow topologies.

CFD simulations can assess the flow topology leading to the particle transport [21,26]. A coupling with an advection model also allows for calculation of Lagrangian tracks and thus a computation of infection risks [27]. However, the quantification of infection risks based on CFD is difficult due to the high number of relevant but often unknown boundary conditions and the wide range of turbulent scales involved. Moreover, the widely used (U)RANS models represent strong simplifications of the actual flow physics due to applied turbulence and Lagrangian dispersion models. It is therefore conceivable that in some situations the results may be distorted due to inadequately modeled mixing and turbulence properties. Therefore, a validation using experimental data, covering large-scale velocity vector fields or particle paths, such as Particle Image Velocimetry (PIV) [28] or Lagrangian Particle Tracking (LPT) [29] is necessary. The experimental approach also allows the inclusion of dynamics and unsteady processes e.g. due to movement of people, which could play a crucial role in mixed convection and thus the local infection risk. However, LPT offers two advantages over PIV for this purpose. On the one hand, the Lagrangian transport routes can be traced directly, which facilitates the interpretation with regard to the probability of infection. On the other hand, the usage of LPT by means of LED illuminated HFSB allows the selection of a significant light sheet thickness in two-dimensional velocity measurements. A conventional 2D-PIV evaluation would cause problems in the cross-correlation scheme due to the crossing flows captured in individual interrogation areas. In this paper we describe a Lagrangian particle tracking experiment, which offers a new perspective regarding aerosol particle transport investigations in closed rooms and allows the flow topologies and transmission paths to be made directly visible in dynamic situations and with different ventilation strategies.

A detailed description of the room, investigated ventilation strategies and the measurement procedure are given in section two. A two-dimensional version of the Shake-The-Box (STB) technique [30] has been used for the identification of particle tracks in a thick LED light-sheet in the front section of the room. With STB, accurate tracking can be achieved, even in densely seeded flows. Since the measurement area covered the entire cross-section of the room, two camera perspectives with an overlapping field of view have been employed. The use of an STB algorithm adapted for application on a two-dimensional field ensures a measurement with high spatial resolution and manageable complexity. The 2D-STB algorithm and evaluation strategy are described in the third section.

This is followed by the visualizations of mean velocity fields and Lagrangian transport paths from potential spreaders to exposed people. To do this, we assume that the influence of respiration on the Lagrangian tracks is negligible. This assumption is justified by experiments [14] and simulations [21] which showed that wearing a mask causes the ejected particles to be transported upwards in the thermal plume of the individual body. Therefore, the transmission path can be evaluated using the Lagrangian particle trajectories, as long as the position of the spreader and the person at risk is known. Particle tracking thus allows a direct assessment of the probability of infection instead of being derived indirectly from the local concentration of aerosol particles.

## 2 Experimental procedure

**Fig. 1:**
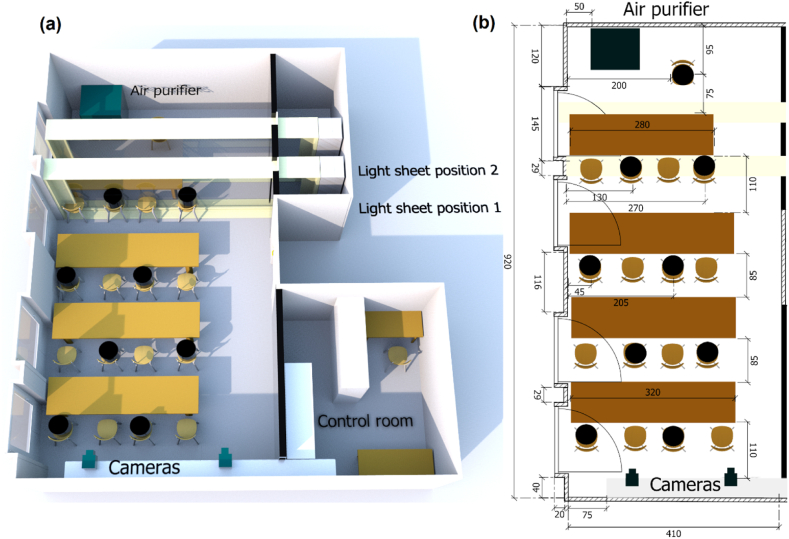
(a) Model classroom at Brandenburg University of Technology (BTU) with (b) length scales and distances in cm. The sketch shows the two light sheet positions used for the present investigation.

A former seminar room at BTU Cottbus-Senftenberg serves as a model classroom. Fig. 1a shows a sketch of the room, which is divided into three segments: the main experimental classroom, the control room yielding necessary technical support and a LED light-sheet support segment. The segments are thermally insulated to prevent the hot air expelled from the measurement equipment from affecting the airflow. The front walls of the room have been covered with black plastic foil and curtain to avoid reflections in the background. The classroom is 2.8 m high and contains 8 students and one teacher modeled by black, cylindrical heated dummies with a volume of 33 L and a surface area exposed to the ambient air of 0.48 m^2^. Each dummy is heated with 75 W and has an almost homogenous surface temperature of approximately 37 °C. All windows are on the left, while the front door is in the middle of the opposite wall. A TROTEC TAC V+ air purifier is located in the front section of the room. Two 2.8 m long pulsed linear LED arrays with collimating lenses developed inhouse by the German Aerospace Center (DLR) Göttingen are used for illumination. The LED arrays are located behind a transparent acrylic glass and create an approximately 40 cm thick light beam, which is reflected back by mirrors on the opposite side. This allows the first row of seats to be illuminated with a 4.2 m × 2.8 m light sheet. Both the LED arrays and the transparent window are movable, so that a volume at the height of the dummies (Light sheet position 1) as well as on the front edge of the first row (Light sheet position 2) of seats can be illuminated. Two Bonito-PRO X–2620 B global shutter CMOS cameras with a resolution of 5120 × 5120 pixels, a pixel size of 4.5 μm and a grey level depth of 10 bit were used to acquire particle images. Each camera was equipped with Nikon lenses with a focal length of 50 mm so that their combined fields of view extended across the entire front row of seats. Image acquisition and pulsed LED illumination were synchronized at 38.5 Hz in order to resolve the Lagrangian particle tracks also in regions with high flow speeds, which is especially important near an open window or an operating air purifier. Due to the large measurement volume, only particles that reflect a particularly high amount of light are feasible. Helium Filled Soap Bubbles (HFSB) meet this requirement due to their mean size of 350 μm and their favorable reflection properties [31,32]. In addition, HFSB are well suited to represent small aerosol particles, because they are almost density neutral and have a low Stokes number [33]. The dimensions of the main experimental classroom are shown in Fig. 1b.

Four scenarios have been investigated each for both light sheet positions.

1. No Ventilation
2. Tilting one window
3. Opening one window
4. Turn on the air purifier

For the last three scenarios additional ventilation was deployed 20 s after starting the measurement. In this way, the transient process caused by the change in boundary conditions was also recorded. The room was seeded for each investigated set of boundary conditions using a HFSB Generator from LaVision GmbH while the nozzles were located in the third bench row. Image acquisition was delayed after seeding until the influence of the HFSB generation on the flow had subsided. By using 130 nozzles simultaneously, sufficient homogenous HFSB density was achieved inside the entire room. The waiting time before image acquisition was 2 min, and although the mean lifetime of the HFSB is limited to approximately 97 s [32] particles-per-pixel (ppp)-values of ∼0.02 per camera imaging the illuminated volume were reached. When calculating the ppp-value, it was considered that only about half of the corresponding camera sensor was employed for tracking. Given the partially non-uniform distribution of HFSB, there is an even greater concentration of particles present in certain areas. Each experiment was performed for 230 s, providing more than 8500 snapshots per time series so that enough data is available to assess the extent to which the average flow has already reached statistically stationary conditions.

Since the change in light-sheet position requires a reconfiguration of the measurement setup, the experiments were performed on two measurement days, resulting in varying temperature differences between the outside and inside temperatures. The temperature difference was approximately −1 K for measurements with the first light sheet position, e.g. illuminating the heated dummies. All measurements using the second light sheet position were conducted with a temperature difference of 5 K. Although it is to be anticipated that the varying temperature differences change the flow, hardly any qualitative effects on the flow topologies under these conditions are to be expected as CFD simulations suggest [34].

## 3 Lagrangian particle tracking using 2D Shake-the-Box

The original STB method [30] is a 3D particle tracking algorithm, relying on the projections of volumetrically illuminated particle clouds onto several cameras, which record the flow in a time-resolved fashion. As a key step, a time-marching predictor/corrector-approach of known particle tracks is used to find the correct solution to the problem of position reconstruction from a few projections, which gets more and more underdetermined the higher the particle image density becomes. For the case considered here - attempting to derive 2D tracks from the projections onto a single camera - the 3D reconstruction problem is absent. However, it is replaced by the problem of correctly identifying particle image peaks in high particle image density conditions and their connections over the time-series. In this ‘2D-STB’ we apply the same methodology as in the 3D-case to leverage the temporal information. A brief explanation of the concept follows; however, a detailed description of the algorithm and its performance is beyond the scope of this contribution and will be published separately.

### 3.1 Peak identification and initial tracking

Particle image peaks are identified using a local maximum criterion with subsequent Gaussian fit. For an increased position accuracy, the found peaks are then optimized using 2D shaking (see below). The peak search is performed on the first four time-steps of a series; from these particle clouds, initial tracks are extracted.

For this, a search radius, corresponding to the expected maximum velocities, is applied to each peak found for time-step 1 (t_1_). The peaks from time-step 2 (t_2_) are queried if they fall within the search radius; if so, a connection (‘track candidate’) is established between the particle from t_1_ and the one from t_2._ This approach is continued for t_3_ and t_4_, always looking for new track candidates and the elongations of already identified ones. Finally, all track candidates of length 4 are identified, the deviation of the positions from a wiener fit [35] is determined and the ones below a certain threshold are accepted as found tracks (basically a maximum acceleration threshold).

### 3.2 Prediction and correction (‘shaking’) of known tracks

Once tracks are known, a predictor/corrector method, being the key step in the STB processing chain, is applied: the already known tracks are elongated (predicted) to the following time-step and the occurring errors are corrected using a position optimization operation (termed ‘shaking’ the reconstructed particles, over progressively decreasing small steps). The position optimization consists of an image matching scheme that compares the image of a single particle, as captured by the camera, with the projected image of the related reconstructed particle. Particle projection requires a shape function, which is sampled around the point-like projection of the particle position to create a virtual particle image. This is done either by a default Gaussian or an optical transfer function (OTF [36]) which was calibrated to the data of this experiment. The cost function is defined for each particle as the difference between original and projected image and is only dependent on the particle position, given a fixed OTF. The gradient of the cost function can be determined numerically [37] or analytically [38], and the particle is moved along several iterations in direction of the steepest gradient. For 3D-STB, the cost function is determined as a sum over all cameras. However, a reduced version of the cost function that operates on a single camera was used here for the 2D-STB.

### 3.3 Convergence and converged phase

The result of the previous step is a cloud of correctly positioned particles, stemming from known tracks. Now, peak detection can be performed on (less populated) residual images and new four-step tracks from time-steps 2–5 are identified. This process is repeated over the full time-series. The self-enhancing interplay of finding more and more tracks and continuously reducing the peaks on the residual image leads to convergence to a state, where only particles newly entering the measurement domain have to be identified, while all others are part of the tracking system and are continued by the predictor/corrector scheme. When available, the extrapolation uses the last 10 timesteps while the last few timesteps have a higher weighting according to wiener filtering theory [39].

Please note that we do not use the velocity information of neighboring particles as predictor or outlier criterion at any time. These approaches can be used quite effectively if 3D positions and velocities are available, however with the applied 2D approach - using only a single camera and a thick light sheet - things are different. As the investigated flow is turbulent, we can observe 3D structures within the light-sheet, leading to different flow regions being projected onto the same image area; therefore, crossing trajectories can occur, which may lead to invalid neighborhood information. Such a situation would also be a major source of uncertainty for correlation-based methods. The predictor/corrector-approach on the other hand treats each particle individually and is therefore able to resolve situations of seemingly crossing trajectories.

### 3.4 Preprocessing and calibration

Before the acquired images were used for evaluation, a sliding minimum image has been subtracted in order to ensure optimal conditions for transient situations with moving windows or people. Because the light was brought in from the right side (from the readers perspective in Fig. 1) and was reflected by an opposite mirror, the intensity as well as the light sheet thickness varies slightly over the cross-section. Therefore, different thresholds for the identification of particles have been used for each camera. The OTF has been calculated in dependence on the position on the camera plane, following the procedure described in Ref. [36]. For this, both camera image planes have been divided into 15 × 15 bins. The shapes of the particle images were determined using a two-dimensional Gaussian fit and averaged for all particles located within a given bin. As a result, astigmatism or blurring due to imaging optics is taken into consideration by the reprojection algorithm, providing a higher accuracy of the shaking process. Calibration from image level to physical level was achieved by a simple measurement of a target with known length. Therefore, errors due to lens distortion and perspective error remain uncompensated. The length distortion read from the calibration image between the edge of the measurement volume and the center indicates an error of ±2.5%. For the maximum perspective error estimation, we presume that the probability density function for horizontal velocity within and velocity perpendicular to the light sheet is roughly equivalent. This leads to a perspective error that rises from the central area of the measurement plane to the periphery, contributing up to 23 mm/s for 99 % of the particles. In addition, the large light sheet thickness causes an error due to the change of the image magnification over depths of approx. ±3,2%..

## 4 Results and discussion

The 2D-STB evaluation of the particle images led to Lagrangian particle trajectories representing 1.5 × 10^5^ to 2.5 × 10^5^ simultaneously tracked HFSB per camera for each experiment at the beginning of the measuring time. The STB-tracking performed individually for each camera requires approx. 10 s of computing on a single AMD Ryzen Threadripper 1950*X* core per time step. Due to the high spatial resolution all relevant scales of the flow in the room were captured. According to the position spectrum of the particle tracks a mean positional accuracy of approximately 150 μm has been reached before applying the temporal wiener filtering which reduces the positional uncertainty down to ∼75 μm. Consequently, this source of error is negligible compared to the systematic errors due to lens distortion and calibration errors because of the large light sheet thickness and perspective effects. In order to get an impression of the influence of the ventilation strategies on the instantaneous and averaged flow topologies, the time-resolved particle tracks, mean velocity fields as well as turbulent kinetic energy fields calculated using a track velocity binning approach are presented in Section 4.1. However, it is difficult to draw conclusions about the probability of disease transmission from these results. To shed light on the transmission paths, we present the Lagrangian tracks that lead through virtual exhalation/inhalation locations above the dummies representing students wearing simple masks. Table 1 shows the abbreviations for all animations presented here.

**Table 1:** Abbreviations for all combinations of animation types (A: animation, F: forward, B: backward) and boundary conditions.

Scenarios	Track Animation	Transport paths forward	Transport paths backward
No ventilation	A1	F1	B1
Window tilted	A2	F2	B2
Window open	A3, A3.2 (dynamic)	F3	B3
Air purifier operating	A4	F4	B4

### 4.1 Flow topologies

The animations A1 to A4 given in the supplementary material show the trajectories of individual HFSB. The tracks on the left side are from the evaluated particle images of the left camera, while the remainder of the measurement volume shows tracks recorded by the right camera. The boundary between the left and right parts of the measuring plane is partially visible. A smoother transition could be achieved by stitching the images from the cameras together before tracking. However, this would require particle matching, which can identify particles in the zone where the FOVs of the cameras overlap despite the different camera perspectives. A 3D STB measurement system with more than three camera projections per sub-volume could solve this open issue.

In the case of no ventilation (A1), it can be seen that the flow is complex and three-dimensional, with particles transported in the thermal plumes reaching vertical velocities of up to approximately 300 mm/s. The animation A2 shows that a tilted window hardly changes anything about the flow topology. However, a fully opened window causes air to flow in lateral direction, which later interacts with the thermal plume of the person sitting next to the window. Animation 4 shows how an air purifier, which is located ∼2.3 m behind the light-sheet and operating with an air exchange rate of 600 m^3^/h, impose a totally different flow topology and increase mixing significantly, especially on the left site of the room. The influence of a moving person is displayed in animation A3.2. Although passing through the light sheet is accompanied by increased mixing and higher velocities, only the thermal plume in direct vicinity is disturbed temporarily. It takes several seconds for the plume to build up again.

As can be seen in the animations, the flow is not stationary, and increases when air exchange and cleaning measures are applied. To quantitatively assess the influence of the ventilation strategy on the stationarity of the velocity field, the turbulent kinetic energy (TKE) $\frac{1}{2}\left(\bar{{v^{\prime }}_{1}^{2}}+\bar{{v^{\prime }}_{2}^{2}}\right)$ computed from both in plane velocity fluctuation components $v_{1}^{\prime }$ (horizontal) and $v_{2}^{\prime }$ (vertical) can be used. It is important to remember that it is not only the turbulence that contributes to the TKE value, but also the large-scale flow changes that occur over longer time scales, as the flow keeps to be transient to some extent. The deviation from statistical stationarity occurs in all cases with ventilation, but especially when the air purifier is used. However, this has little effect on the ability to interpret the TKE as a measure of mixing intensity, as the large-scale flow changes also contribute to mixing. Table 2 and Table 3 show how the mean TKE computed from all velocity bins actually increases with the ventilation.

**Table 2:** Global average of TKE contributions for each ventilation strategy lightsheet position 1 in mm^2^/s^2^.

Scenario (LS1)	No Ventilation	Window tilted	Window open	Operating purifier
$\frac{1}{2}\bar{{v^{\prime }}_{1}^{2}}$ (horizontal)	351.8	560.1	564.6	2154.7
$\frac{1}{2}\bar{{v^{\prime }}_{2}^{2}}$ (vertical)	660.3	747.8	917.4	2328.7
Global TKE	1012.1	1307.9	1482.0	4483.4

**Table 3:** Global average of TKE contributions for each ventilation strategy at lightsheet position 2 in mm^2^/s^2^.

Scenario (LS2)	No Ventilation	Window tilted	Window open	Operating purifier
$\frac{1}{2}\bar{{v^{\prime }}_{1}^{2}}$ (horizontal)	71.6	373.9	626.8	2389.3
$\frac{1}{2}\bar{{v^{\prime }}_{2}^{2}}$ (vertical)	76.3	395.1	503.5	2409.5
Global TKE	147.9	769.1	1130.3	4798.8

The largest impact onto the TKE is reached by operating the air purifier, which is in close proximity to both light sheet positions. Both opening and tilting the window significantly increases the TKE, with opening the window having the greater effect, as expected. This observation applies to both light sheet positions. However, a comparison of the results provided in Table 2, Table 3 shows that the TKE contributions of vertical velocity are higher than the contribution of horizontal velocity due to the presence of thermal plumes in the first light sheet position, which is not the case for the results of light sheet position two. Opening or tilting the window increases both the horizontal and vertical variance in both cases.

**Fig. 2:**
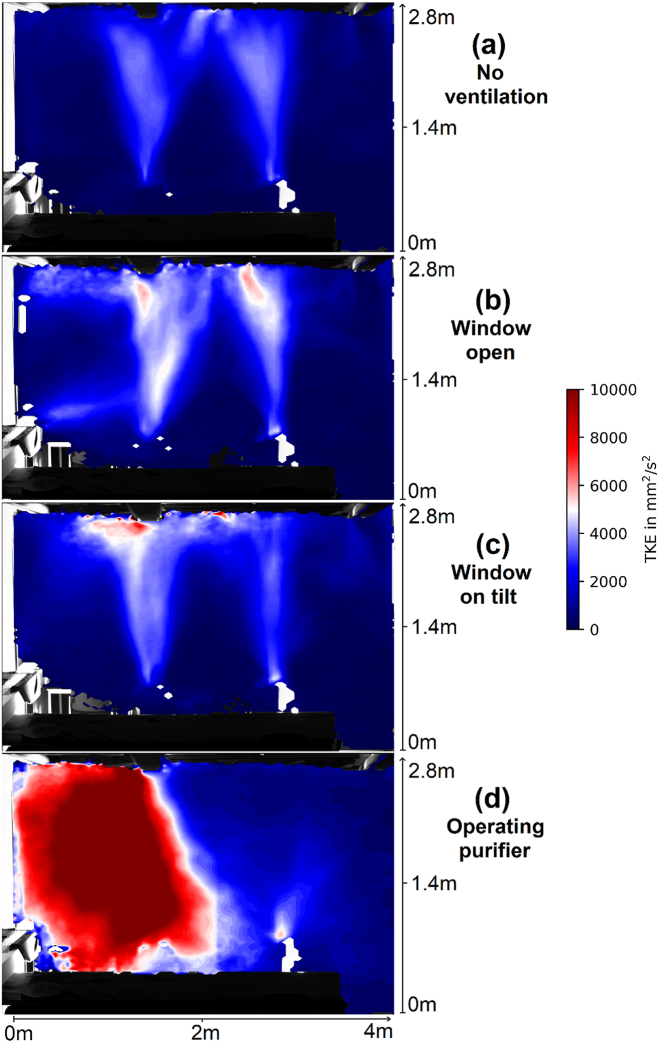
Turbulent kinetic energy (TKE) considering both in-plane velocity components at lightsheet position 1 for a scenario (a) without any ventilation, (b) with an open window (c) with a tilted window and (d) with an operating air purifier.

Fig. 2 shows the local distribution of the TKE for light sheet position 1. The plots in Fig. 2b and c shows that opening or tilting the window increases the TKE in the plumes and creates new regions of increased TKE, in contrast to the unventilated scenario demonstrated in Fig. 2a. These new regions extend not only in the immediate vicinity of the open window but also in the upper region on the wall side. This indicates a higher degree of mixing even in distant positions in space, which remained hidden in the animations A2 and A3. Fig. 2d shows a significantly higher TKE on the left side, in contrast to the three cases without active air purifier. In addition, the source of TKE is no longer the thermal plumes but the air purifier.

**Fig. 3:**
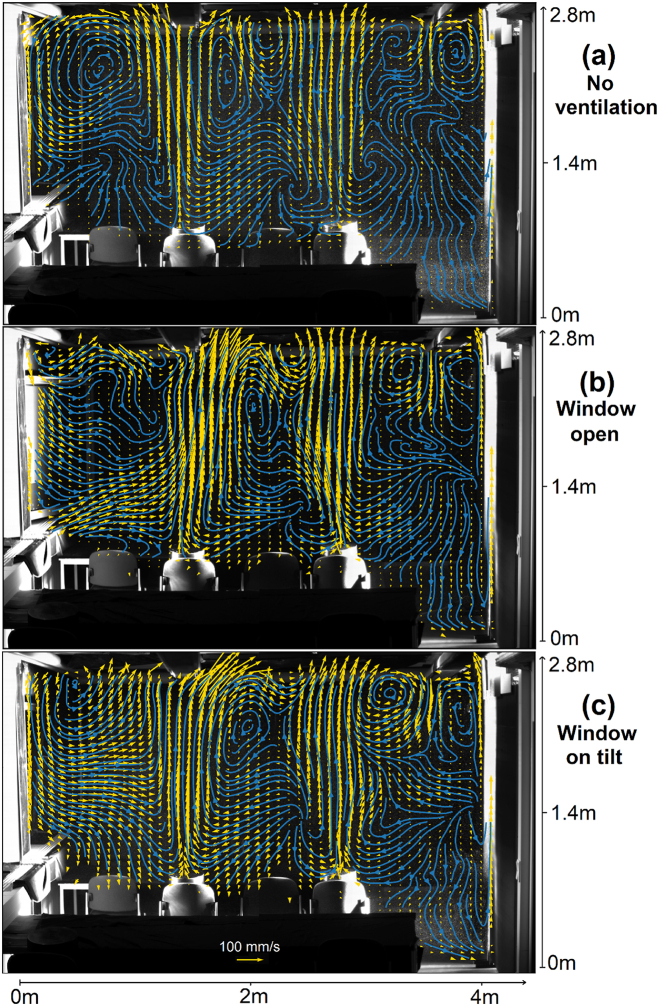
Mean velocity field (yellow) and respective Eulerian streamlines (blue) at lightsheet position 1 for a scenario (a) without any ventilation, (b) with an open window and (c) with a tilted window. The temperature inside the room is 1 K lower than outside temperature. (For interpretation of the references to color in this figure legend, the reader is referred to the Web version of this article.)

Furthermore, the prevailing mean large-scale velocity topologies are critical in determining how the aerosol is transported and thus whether pathogens are carried directly from one infected person to the next. These velocity fields measured at the same light-sheet position as for the track animations are shown in Fig. 3. It can be seen in Fig. 3a, that in the case of no ventilation the convective heat transfer dominates the flow. The location of recirculation areas seems to be influenced by the lamps on the ceiling. It was reported earlier [40], that lamps on the ceiling divide the flow imposed by an air purifier into two regions reducing the cleaning efficiency. The fact that even the position of the recirculation areas created by the thermal plumes depends on the position of the lamps shows that the room geometry, even without ventilation, has a major impact on the potential transmission paths. Opening or tilting a window (Fig. 3b and c changes the flow topology only in its direct vicinity, although over several minutes of opening, still a significant air exchange and thus reduction of aerosol particles can be reached [24].

**Fig. 4:**
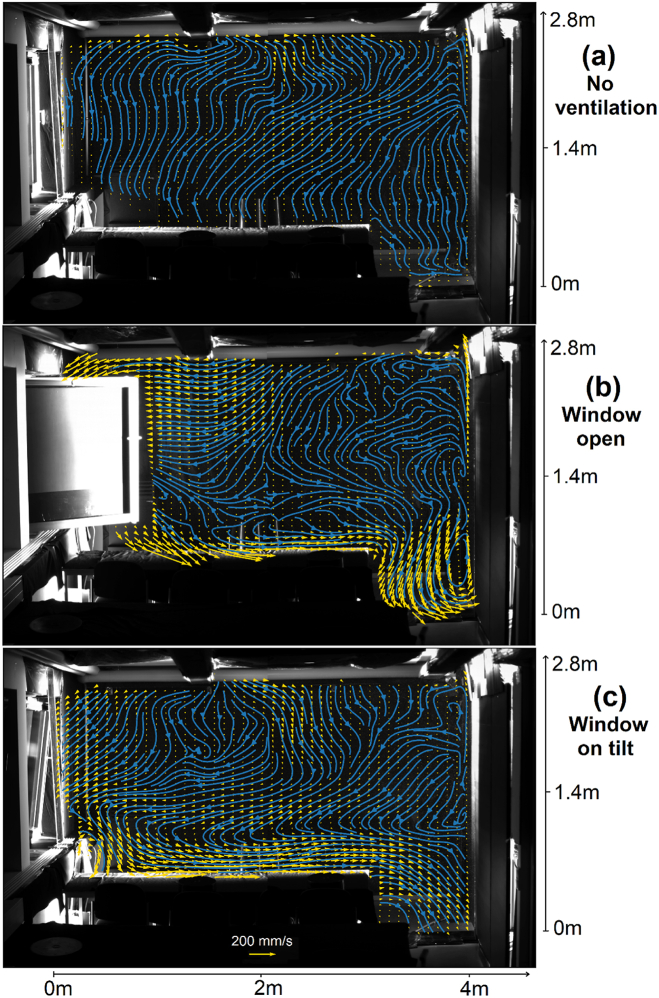
Mean velocity field (yellow) and Eulerian streamlines (blue) at light-sheet position 2 for a scenario (a) without any ventilation, (b) with an open window and (c) with a tilted window. The temperature inside the room is 5 K higher than outside temperature. (For interpretation of the references to color in this figure legend, the reader is referred to the Web version of this article.)

The flow at the second light sheet position, i.e. above the front side of the desks is shown in Fig. 4. In a scenario without ventilation (Fig. 4a), the air moves downwards indicating a recirculation area in between the seat rows. Furthermore, Fig. 4c and particularly Fig. 4b show that the different buoyancy forces in the thermally stratified room air influence the pressure field in such a way that suction from the center of the room is favored. Furthermore, the colder air flowing in from the outside sinks to the table and move towards the opposite wall. The window opening thus creates a horizontal circulation that allows some air exchange even from the opposite wall, at least in a plane orthogonal to the open window. A joint analysis of the results from both light sheet positions show that the plumes remain when the window is opened, but the lateral recirculation areas almost disappear (Fig. 3, Fig. 4b). This does not happen when the window is only tilted, as can be seen in Fig. 3, Fig. 4c. The recirculation between the rows of seats remains in both cases to a certain extent, but is superimposed by the additional horizontal circulation that counteracts thermal stratification. However, it should be noted in this interpretation that the difference between outside and room temperature was different for the measurements with different light sheet positions. The animations A2 and A3 give an impression of the time scales over which the topology changes occur.

**Fig. 5:**
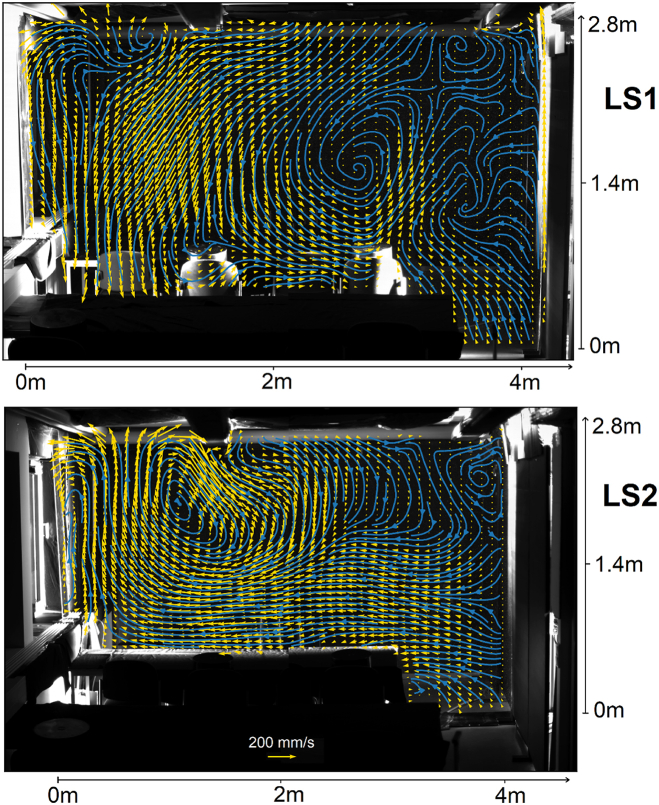
Mean velocity field (yellow) and Eulerian streamlines (blue) with air purifier operating at 600 m^3^/h (top: lightsheet position 1, bottom: lightsheet position 2). (For interpretation of the references to color in this figure legend, the reader is referred to the Web version of this article.)

The influence of the air purifier is the strongest, as can be seen in Fig. 5 and Animation A4. The lateral recirculation, plumes as well as the recirculation in between the seat rows became completely three-dimensional. The flow velocities are increased significantly, which leads to increased mixing of aerosol particles. However, this result is consistent with the findings of an experiment using saliva particles carried out in the same room and air purifier position [24]. It has been shown that the use of the purifier in a continuous spreading situation quickly leads to a stable concentration of particles in the middle of the room, which suggests strong mixing even in distant locations. Nevertheless, the average velocities at the positions 1.1 m above the chairs on which the dummies are placed remain calm. A quantitative comparison shows that the velocities at these positions are even higher without ventilation (64 and 83 mm/s) than with ventilation via an operating air purifier (61 and 52 mm/s). Therefore, the altered airflow is not expected to affect comfort.

In summary it can be concluded, that the plumes and recirculation topologies get disturbed by all of the investigated ventilation strategies. However, the effect of an open or tilted window on the thermal plume on the right site of the room is low. Open windows nevertheless lead to a decrease in aerosol concentration even at remote positions [24]. A considerably higher influence on the flow topology is found when an air purifier is operating. In this case, the flow on both sides is changed and turbulent mixing which prevents local and global particle accumulation, is increased.

### 4.2 Lagrangian tracks of exhaled and inhaled particles

Aerosol particles exhaled by infected persons can lead to infections in different ways Firstly, the concentration in the room can increase due to the continuous exhalation of virus-laden aerosol particles, so that all those who are in the same room for a long time are at risk of infection. Secondly, infections can also be caused by aerosol particles that are transported directly from person to person, as described in Ref. [19]. This section examines how ventilation strategies affect transport routes to draw conclusions about the risk of direct infections. Therefore, particle tracks which pass through a virtual exhalation location (see animations F1 to F4) above the dummies were gathered over multiple timesteps. The point in time when the track is in one of those specified virtual exhalation locations has been redefined as the common start of all tracks, so that many tracks leave the rectangular area simultaneously. The search was performed in four time slots, each consisting of multiple time instants, such that four “exhalations” can be seen in the animations. The color of the particles shown indicates the virtual exhalation position from which they originate. The stronger the blue or red color, the faster the particle is moving.

Based on the assumption that aerosol particles exhaled by a mask-wearing individual follow the thermal plume [13,14] the tracks shown in animations F1 to F4 are expected to represent the transport of virus laden particles exhaled by an infected person.

It should be noted that although the field of views of the cameras overlap, each camera depicts only one dummy completely. Therefore, even with a direct flow from one dummy to the next, the tracks end near the edge of the other dummy. In addition, only those particles are considered which do not leave the light sheet. Since the flow has a non-negligible component orthogonal to the light sheet as discussed in section 4.1, many particles leave the light sheet during the measurement time. Therefore, the length of the particle tracks is limited, so the animations presented in the supplementary material show only ∼20 s s long tracks. Nevertheless, if direct transport between the location of the dummies exists for any ventilation strategy, the measurement setup is able to detect it due to the small distance between the dummies.

Animation F1 shows how the particles are transported to the ceiling, when there is no disturbance due to ventilation. Direct infections of persons in the same row by aerosols are not to be expected in this case. An open or tilted window does not have a qualitative impact on the transport path, except for a slightly increased distribution of particles along the plume on the left side (Animation F2, F3). This suggests that in the short term, despite the expected thermal stratification in the classroom, there is little air exchange to the right of the left dummy through the window opening. Instead, a zone is created in the left part of the room in which the fresh air mixes with the room air. This interpretation is also supported by the mean airflow shown in Fig. 3. With an operating air purifier, the particles exhaled by the left virtual spreader are distributed much further, while there is a comparably small qualitative difference observable on the right site (Animation F4). There is no direct flow from one person to the next in this case, too. Note that the Eulerian streamlines obtained from the mean flow shown in Fig. 5 do not represent the Lagrangian transport observed which shows the highly unsteady behavior in the vicinity of an active air purifier.

Another perspective to study the transport of aerosols is provided by depicting tracks of particles ending at positions where they get inhaled. For this purpose, all particle trajectories ending 1.1 m above the dummies seats were displayed in the animations B1 to B4 as if they would start simultaneously at their respective initial positions.

The animations thus show from which regions the particles originate that would be inhaled by the respective dummy in a real spreading situation. The trajectories shown in B1 to B4 support the observations from F1 to F4 that none of the ventilation measures lead to a direct flow from the area above one dummy to the next dummy. Thus, all ventilation strategies investigated do not lead to an increased risk of direct infection by aerosol particles transported on a short, lateral path between the persons. The results demonstrate also the differences between Eulerian streamlines based on time averaged velocity fields and the instantaneous Lagrangian tracks. Consequently, the route of transmission of viruses from an infected to a susceptible person deviates from Eulerian streamlines.

## 5 Conclusions and future work

The presented results show the potential of Lagrangian particle tracking of HFSB in large volumes to examine aerosol particle transport in enclosed spaces. This approach enables a new perspective especially in dynamic situations, which are not covered by simulations. The instantaneous flow animations show that the time scales of flow topology change after opening/tilting a window and the dynamic induced by people passing the light sheet.

In addition, the effect of different ventilation strategies on aerosol particle transport was investigated to determine whether they are suitable for preventing indirect infections. The results appear to be not completely stationary, but characteristic features of the flow topology can still be found for each ventilation strategy. Each scenario was measured for approx. 3 min at a high seeding density despite the limited lifetime of HFSB. In spite of these limitations, the data analysis leads to the following conclusions regarding the influence of ventilation strategies on the flow topology and aerosol particle dispersion.

1. The plumes of the students create lengthwise circulating vortices, which cannot be destroyed across the entire cross-section of the room by opening or tilting a window, when temperature difference to the outside air is small.
2. The mixing in the room through the air purifier at the given flow rate is higher compared to opening a window, which suggests that this strategy in combination with its air filtering capability is the most effective strategy (see also [24]). However, the flow generated by an air purifier is tightly linked to its position in the room, particularly to its proximity to a wall.
3. The risk of infection due to a directed lateral flow of exhaled, virus-laden aerosol particles is relatively low in any of the ventilation methods examined.

It should be noted that the latter conclusion only applies to possible infections caused by aerosols up to about 5 μm in diameter, which follow the flow as almost passive tracers. However, heavier and larger particles are filtered by conventional masks more efficiently, reducing the infection risk. Furthermore, the windows in the model classroom are not located directly next to the students, which may have a big influence. Moreover, it is recommended that validation using other measurement techniques be undertaken as the outcomes presented in this investigation are derived solely from the results of a single measurement technique.

All three findings apply for only half occupied seat rows, which was one of the preventive measures in schools during the first COVID-19 pandemic. Whether there are qualitative changes to this pattern with full rows of seats must be clarified by future experiments. Another additional aspect of major importance worth investigating concerns the influence of radiators on the flow topology. To shed light on these issues, a volumetric experiment using 3D LPT would be necessary, which is also appropriate to answer some of the remaining questions raised in this contribution.

At first, it could be examined in detail how the particles are transported from one row to the next within the spanwise circulation. Secondly, it would allow a determination of the probability of infection for all seat locations in the measurement volume, assuming a known rate of exhaled viruses entrapped in aerosol particles for a single spreader. A three-dimensional tracking dataset will also allow validation of CFD methods. However, the illumination and recording of a spacious room is challenging. The first challenge is where to place the required large number of pulsed LEDs and how to prevent the influence of waste heat on the measurement. In the second place, the apertures of the camera lenses have to be closed, in order to ensure sharp imaging of the particles from different volume depths, which will further decrease the available light budget. However, these problems can be solved by a scanning approach in which sub-volumes are illuminated one after the other.

## Ethics statement

Review and/or approval by an ethics committee and informed consent was not required for this study, as no participants not involved in the preparation of the manuscript were required for the experiments performed.

## Data availability statement

The data has not been deposited in a publicly accessible repository, but will be made available upon reasonable request.

## CRediT authorship contribution statement

**Tom Buchwald:** Writing – review & editing, Writing – original draft, Visualization, Validation, Methodology, Investigation, Formal analysis, Data curation, Conceptualization. **Gazi Hasanuzzaman:** Writing – review & editing, Supervision, Investigation. **Sebastian Merbold:** Writing – review & editing, Supervision, Project administration. **Daniel Schanz:** Writing – review & editing, Software. **Christoph Egbers:** Writing – review & editing, Supervision, Project administration, Funding acquisition. **Andreas Schröder:** Writing – review & editing, Supervision, Software, Resources, Project administration, Methodology, Funding acquisition, Formal analysis.

## Declaration of competing interest

The authors declare that they have no known competing financial interests or personal relationships that could have appeared to influence the work reported in this paper.

## Appendix A Supplementary data

Multimedia components A1-A4, F1-F4, B1-B4 Multimedia components A1-A4, F1-F4, B1-B4

Multimedia component A1 Multimedia component A1

Multimedia component A2 Multimedia component A2

Multimedia component A3.2 Multimedia component A3.2

Multimedia component A3 Multimedia component A3

Multimedia component A4 Multimedia component A4
