# Supplementary figures and images for: Large-scale flow field and aerosol particle transport investigations in a classroom using 2D-Shake-The-Box Lagrangian Particle Tracking
Source: Heliyon. 2023 Nov 27;9(12):e22826. doi: 10.1016/j.heliyon.2023.e22826 (PMC10704378; doi:10.1016/j.heliyon.2023.e22826)

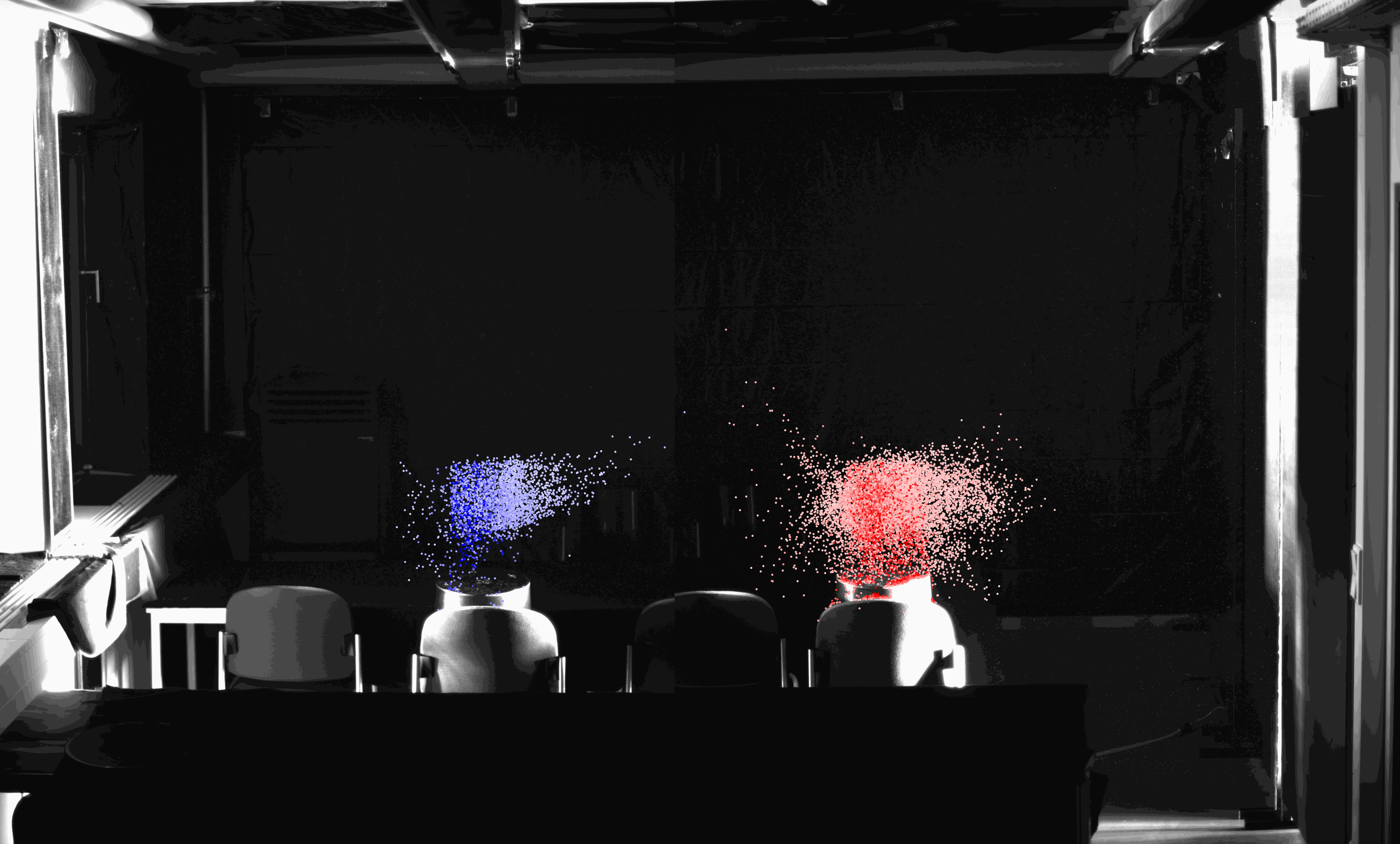

Supplement: Multimedia components A1-A4, F1-F4, B1-B4 [file mmc1.zip › Transport_Paths/B1_reference.gif]

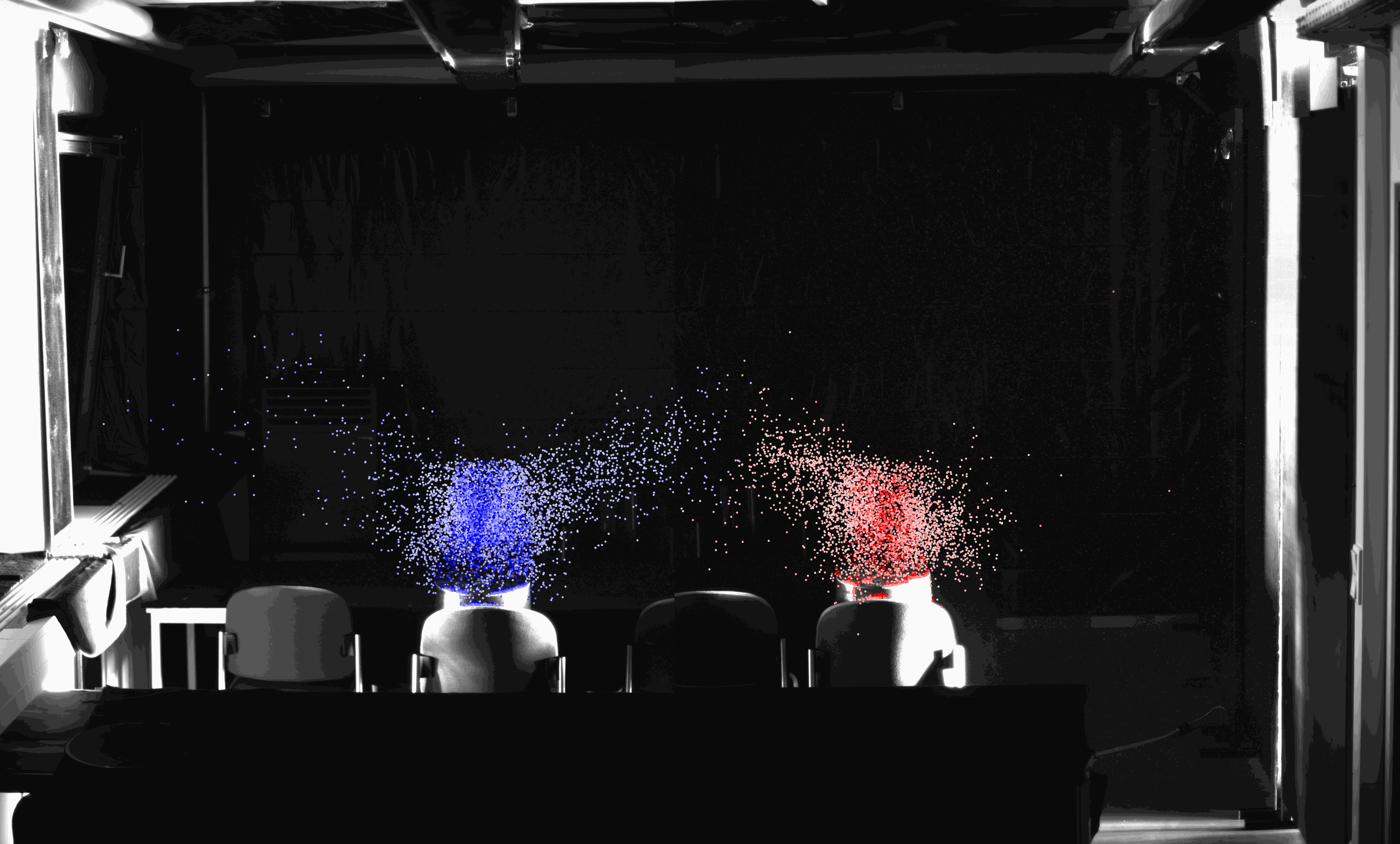

Supplement: Multimedia components A1-A4, F1-F4, B1-B4 [file mmc1.zip › Transport_Paths/B2_tilt.gif]

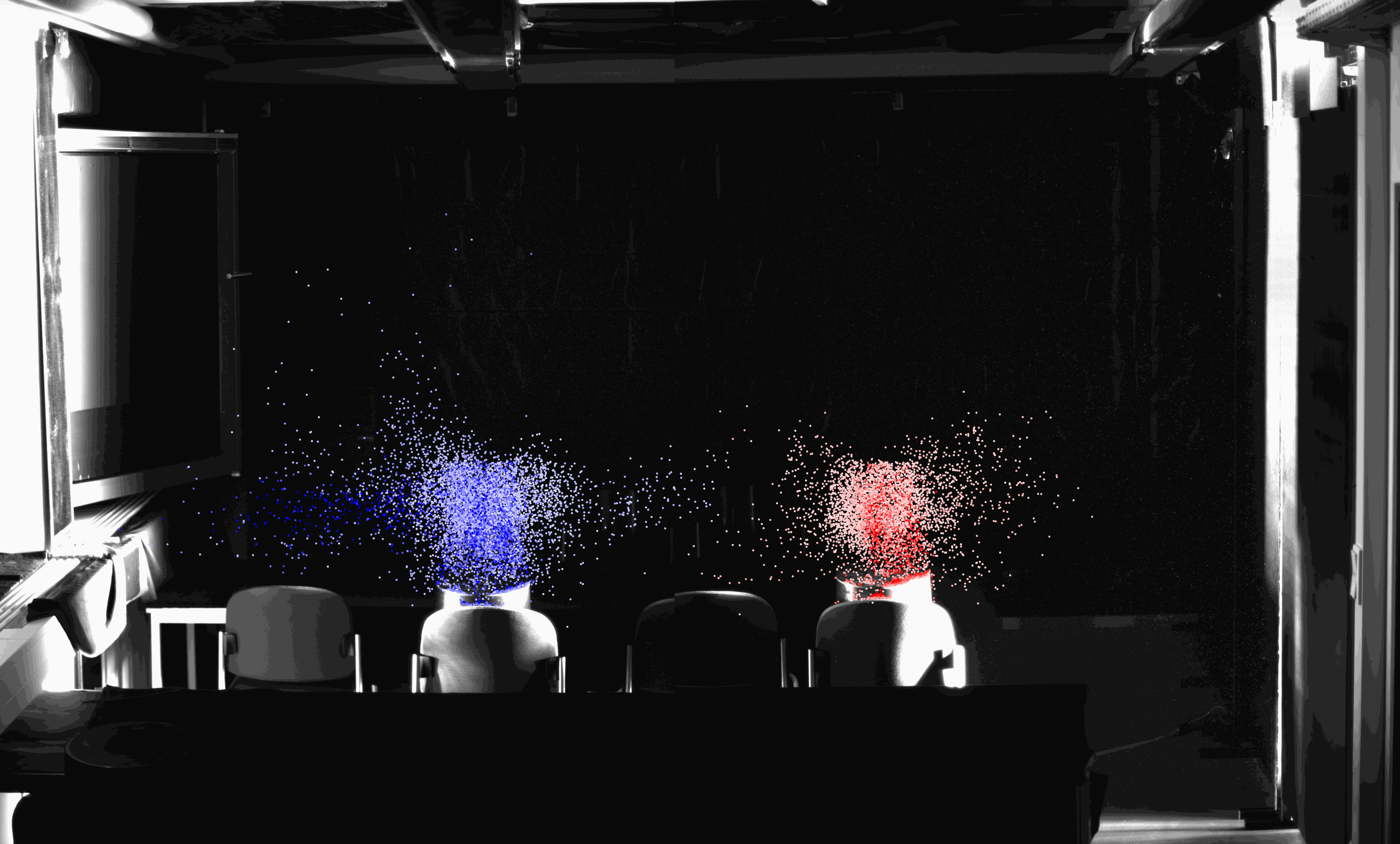

Supplement: Multimedia components A1-A4, F1-F4, B1-B4 [file mmc1.zip › Transport_Paths/B3_open.gif]

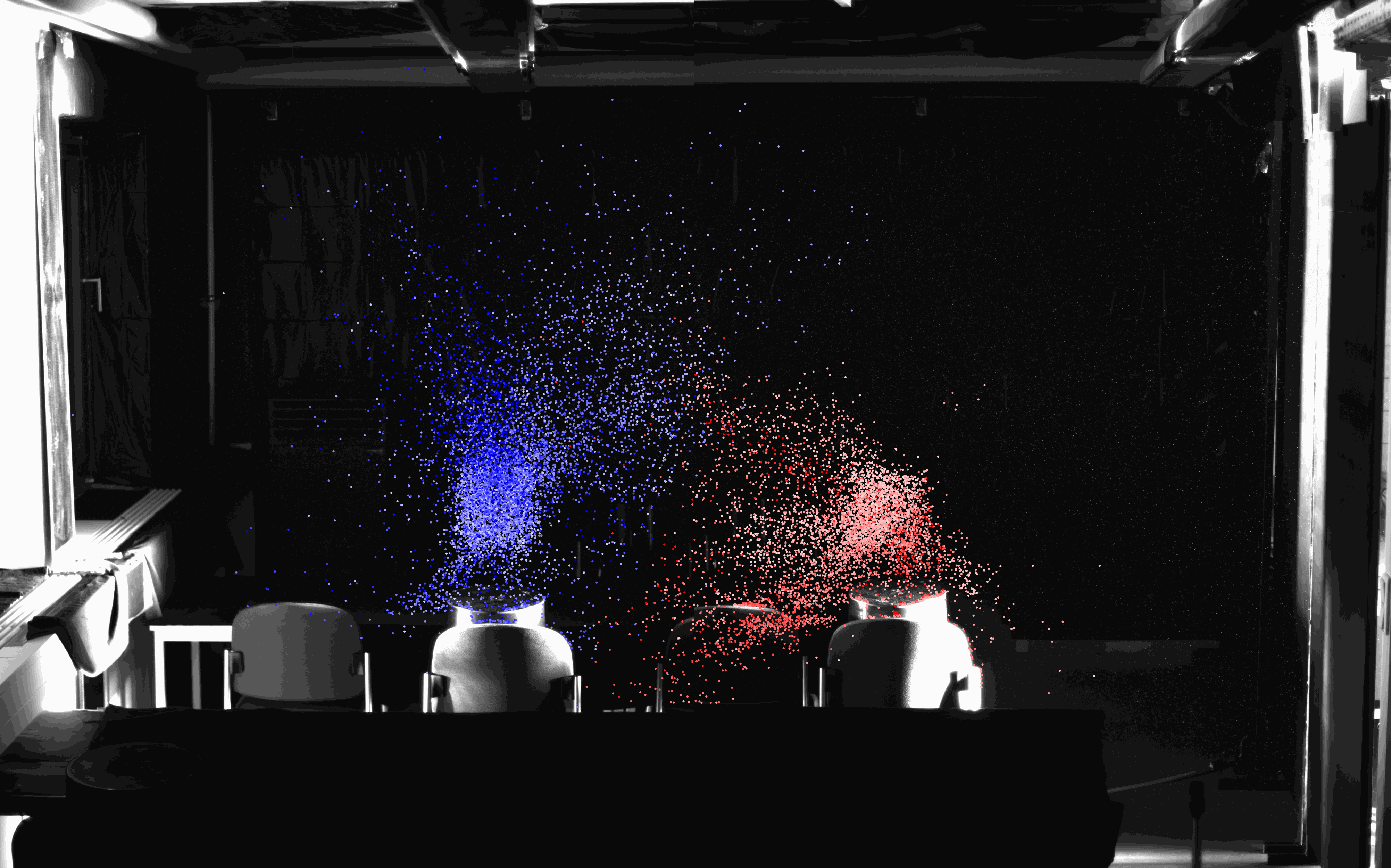

Supplement: Multimedia components A1-A4, F1-F4, B1-B4 [file mmc1.zip › Transport_Paths/B4_purifier.gif]
